# China data on the burden of alcohol-associated liver cancer from 1990 to 2021: a systematic analysis from the global burden of disease study 2021

**DOI:** 10.3332/ecancer.2026.2057

**Published:** 2026-01-13

**Authors:** Nan Luo, Prasai Arzoo, Juanli Xua, Pengkhun Novb, Jiqiang Li

**Affiliations:** 1 Department of Radiation Oncology, Oncology Center, Zhujiang Hospital, Southern Medical University, No. 253 Mid Gongye Avenue, Haizhu District, Guangzhou, Guangdong Province 510282, China; a https://orcid.org/0000-0002-585-5911; b https://orcid.org/0000-0002-7016-8285

**Keywords:** GBD, alcohol-related liver cancer (A-LC), mortality, disability-adjusted life years, epidemiology

## Abstract

**Background:**

Alcohol consumption is a significant risk factor for liver cancer, particularly hepatocellular carcinoma. In China, the incidence of liver cancer has been rising, necessitating an in-depth analysis of the relationship between alcohol consumption and liver cancer burden.

**Objective:**

This study aims to assess the burden of alcohol-associated liver cancer (A-LC) in China from 1990 to 2021, utilising data from the Global Burden of Disease (GBD) Study 2021.

**Methods:**

This study first gathered data on A-LC in China, focusing on age, sex, incidence, prevalence, mortality, disability-adjusted life years (DALYs) and risk factors, using information from the 2021 GBD study covering the years 1990–2021. Next, the research examined the temporal trends of A-LC burdens in China during the same period, employing linear regression modeling to calculate estimated annual percentage change (EAPC) values. Additionally, the autoregressive integrated moving average and exponential smoothing model were utilised to project future disease burdens from 2022 to 2050. Finally, the study analysed risk factors associated with A-LC.

**Results:**

The number of deaths and DALYs for A-LC grew significantly during the study period 1990–2021 (in 1990, in 2021). However, age-standardised deaths and DALYs declined (deaths: 0.87 in 1990 and 0.85 in 2021, DALYs: 24.26 in 1990 and 22.01 in 2021). EAPC = 0.16 (95% CI 0–0.32) for deaths in patients with A-LC and EAPC = −0.18 (95% CI −0.34 to −0.01) for DALYs. Although age-standardized death rates have declined in the last 3 years, the number of incidence, disease and death cases of A-LC patients in China has increased with the progress of time. In 2021, ASIR and ASPR reached the highest in history and the large base of A-LC patients in China and the overall situation of the people should not be underestimated. While East Asia and the high-income Asia-Pacific region declined over the study period, Central Asia saw an increase in age-standardised DALY rates. Age-standardised DALYs also grew significantly in high-income populations in North America and Australasia.

**Conclusion:**

A-LC remains a serious threat to the major health problems of the Chinese people, especially men, and the burden of liver cancer associated with obesity risk factors is also increasing significantly and is expected to continue to grow over the next 25 years.

## Introduction

Liver cancer, particularly hepatocellular carcinoma (HCC), has emerged as a significant global health burden, with alcohol consumption identified as a major contributing factor [1]. In recent decades, China has witnessed a marked increase in liver cancer incidence, paralleling rising alcohol consumption rates [2, 3]. Alcohol-associated liver disease encompasses a spectrum of conditions, including fatty liver, alcoholic hepatitis and cirrhosis, which can predispose individuals to liver cancer [4–6].

Previous studies analysing the burden of primary liver cancer in the literature have focused on the distribution of countries or regions globally, on a single most common cause, such as Hepatitis B virus and Hepatitis C virus (HCV) infection or alcohol consumption or on single-year studies that did not analyse overall time trends [7–11]. However, information focusing on the Chinese region is still lacking in up-to-date analyses. The only literature available in the repository showed that in China, the number of primary liver cancer cases and deaths grew to 433.11% from 1919 to 1990 and 42.68% from 1990 to 2019. From 1990 to 2019, the global age-standardised incidence rate (ASIR) and age-standardised rate (ASR) mortality rate (ASMR) of primary liver cancer declined on average each year, whereas from 1990 to 2019, the ASIR of primary liver cancer and the ASMR showed a stable trend [12]. Between 1990 and 2019, nearly half of the countries had an upward trend in ASIR for primary liver cancer, and more than one-third of the countries supported an upward trend in ASIRs for primary liver cancer of all etiologies globally [12, 13].

The Global Burden of Disease (GBD) Study provides comprehensive data on disease prevalence, incidence, mortality and risk factors across countries and regions. Understanding the burden of alcohol-associated liver cancer (A-LC) in China from 1990 to 2021 is crucial for public health planning and intervention strategies. In this study, we aim to: (1) Describe China Data on the Burden of A-LC from 1990 to 2021; (2) Identify temporal trends in A-LC burdens; (3) Quantify the contribution of risk factors to A-LC incidence, death, prevalence and DALYs in china; (4) Develop predictive models to project future A-LC burdens in china from 2022 to 2050. The main goal of this study is to illuminate epidemiologic patterns in order to guide resource allocation and track progress toward achieving health-related Sustainable Development Goals.

## Methodology

### Materials and methods

#### Overview

**GBD source:** The 2021 GBD analysis synthesises contemporary epidemiological data through standardised protocols to assess disease burden across 204 countries and territories. This comprehensive investigation examines 371 health conditions and their associated impairments, along with 88 risk factors. Results are accessible via the GBD Results Tool (http://ghdx.healthdata.org/gbd-results-tool), which facilitates data stratification by demographics and 27 geographical regions. To assess the burden of A-LC in China, we retrieved data from the Global Health Data Exchange (https://ghdx.healthdata.org/gbd-resultstool).

For tracking A-LC epidemiology, we employed two key metrics: ASRs and estimated annual percentage changes (EAPCs). ASR enables the identification of disease patterns within populations, informing targeted prevention strategies, while EAPC quantifies temporal trends in disease burden across demographic groups.

Methodologically, we utilised DisMod-MR 2.1, implementing Bayesian meta-regression through a systematic framework. Data preparation involved disaggregation of non-specific demographic information and application of Meta-Regression-Bayesian, Regularised, Trimmed techniques to ensure cross-study comparability.

### Statistical analysis

Our analysis presents a comprehensive assessment of A-LC burden, examining 2021 data for incidence, prevalence, mortality and disability-adjusted life years (DALYs) at Chinese scales. We stratified results by demographic factors, including age and sex, enabling the identification of population-specific disease patterns and evaluation of intervention efficacy.

Temporal trend analysis spanned 1990–2021, employing linear regression to calculate EAPCs in ASRs. We defined trend directionality using 95% uncertainty intervals (UIs): increasing trends required positive EAPC estimates with lower UI bounds above zero, while decreasing trends required negative estimates with upper UI bounds below zero. Cases falling between these criteria were classified as stable.

Risk factor analysis emphasised secondary and tertiary determinants, examining mortality and DALYs associated with Level 2 factors (alcohol and smoking) and Level 4 factors (body mass index). This approach aligns with World Cancer Research Fund guidelines, employing comparative risk assessment frameworks. Complete risk factor methodologies are available in the 2021 GBD risk factors report.

For future trajectory modeling, we implemented AutoRegressive Integrated Moving Average (ARIMA) analysis. This approach necessitated data stationarity through differencing procedures. Model parameters (p,d,q) were optimised using autocorrelation (ACF) and partial ACF functions (PACFs). Forecasting utilised R software (version 4.3.2) with forecast and series packages. Model validation incorporated multiple statistical tests: Ljung-Box Q-test for forecast error independence, Shapiro-Wilk test for residual normality and Breusch-Pagan test for homoscedasticity. Furthermore, we also use the exponential smoothing (ES) model to predict. ES is a forecasting technique that leverages past observations to predict future values. It excels in handling time series data, where observations are recorded at consistent intervals. ES assigns higher significance to recent observations, while retaining consideration for older data, allowing it to readily adapt to evolving patterns. Statistical significance was established at *p* < 0.05.

## Result

### 2021 China burden of A-LC

**Figure 1.**
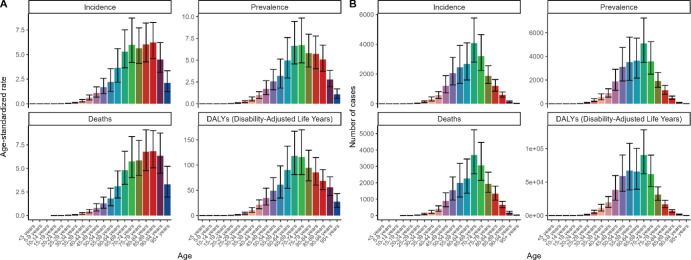
Visualises the trend of ASR and a number of cases for different age groups regarding incidence, prevalence, mortality and DALYs in China in 2021. (A) ASR of cases for different age groups regarding incidence, prevalence, mortality and DALYs. (B) A number of cases for different age groups regarding incidence, prevalence, mortality and DALYs.

From the Figure 1, we can see that in terms of A-LC incidence and a number of diseased cases, A-LC in China is mainly concentrated and highly incidence in the elderly aged 60–74 years, especially in the age group of 65–69 years and the number of A-LC patients in China in 2021 reached a maximum of 4,072 (95% UI, 2,796–5,762) cases; whereas, A-LC rarely occurs in the middle-aged and young people younger than 40 years of age (Figure 1b, Table 1).

Observation of ASIRs, prevalence rates, mortality rates and DALYs of patients with A-LC that the trends of these four major indicators were slightly different in each age group (Figure 1a). In terms of ASIR, in patients younger than 74 years of age, ASIR grew yearly with age and declined in the age group of 75–79 years, then increased to reach the maximum value of ASIR in the age group of 85–89 years. Mean 6.22 (95% UI, 4.61–8.27) per 100,000 and then decreased with age (Figure 1a, Table 1).

Age-standardised prevalence rates (ASPRs) and age-standardised death rates (ASDRs) followed essentially the same trend, sharing the common pattern of growing to a peak and then declining with age, with the difference that the peak ASPR of 6.72 (95% UI, 4.68–9.84) per 100,000 occurred in the 70–74 year age group, whereas the peak ASDR of 6.82 (95% UI 5.05–9.02) occurred in the 85–89 age group (Figure 1a, Tables 2 and 3).

The overall trend in age-standardized disability-adjusted life-years rate (ASDAR) change also showed an increase and then a decrease with age, with the peak age groups of 65–69 and 70–74 exceeding 100 in both phases of the data, reaching 118.19 (95% UI, 81.32–167.08), 116.17 (95% UI, 81.32–169.75) per 100,000 population (Figure 1a, Table 4).

In conclusion, the overall trends of ASR and number of the four major epidemiological indicators of A-LC patients in China were similar, with the high prevalence of the disease concentrated between 65 and 89 years of age, although there were slight differences in the age groups in which the maximum values of the respective indicators were located and the ASR and number of cases for the four major indicators rose before the period of concentrated high prevalence and then declined after the period of concentrated high prevalence (Figure 1).

In addition, the ASRs associated with A-LC incidence, prevalence and death were almost zero in young and middle-aged people before the age of 40 years, indicating that the probability of alcoholic liver cancer in young and middle-aged people in China is very low; and the population DALYs were also much smaller than 11 (Figure 1, Table 4).

**Figure 2.**
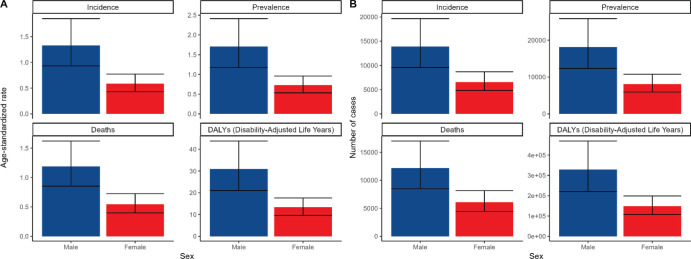
Shows the comparison of ASRs and number associated with incidence, prevalence, death and DALYs of A-LC patients of different genders in China in 2021.

In 2021, the number of A-LC incidence in China was 13,885 (95% UI, 9,569–19,671) for males and 6,579 (95% UI, 4,820–8,697) for females, with an ASIR of 1.33 (95% UI, 0.93–1.85) per 100,000 for males and 0.58 (95% UI, 0.43–0.77), with the number of cases and ASIR in men 2.11 and 2.29 times higher than those in women, respectively (Figure 2, Table 1). In 2021, the number of A-LC cases and deaths in Chinese men were 18,117 (95% UI, 12,306–25,853) and 12,198 (95% UI, 8,489–17,015), respectively, which were 2.23 and 6,119 (95% UI, 4,472–8,144) times higher than the corresponding figures for women (8,125 (95% UI, 5,909–10,777) and 6,119 (95% UI, 4,472–8,144) 2.23 and 1.99 times (Figure 2, Tables 2 and 3).

In the case of DALYs, despite the decrease in both male and female ASDAR in 2021 compared to the previous 1990, the value of ASDAR in 2021 was still significantly higher for males 30.96 (95% UI, 21.08–43.77) than for females 13.34 (95% UI, 9.6–17.61) and males were 2.32 times more likely to be female (Figure 2, Table 4). The male EAPC was 0 (95% CI, −0.16 to 0.17) and the female EAPC was −0.46 (95% CI, −0.61 to −0.3) (Table 4).

The overall data for males exceeded those for females by a significant multiplicative relationship, both in terms of liver cancer incidence, prevalence, deaths, ASRs associated with DALYs (Figure 2a), and in terms of the number of cases for the four major epidemiological indicators (Figure 2b) (Figure 2). Such a clear trend of gender difference in the data in Figure 2 indicates that men are at high risk of developing A-LC in China.

*Comparison of changes in the four main indicators of incidence, prevalence, mortality and DALYs of A-LC 1990–2021 and interpretation of their underlying etiological analyses*

**Figure 3.**
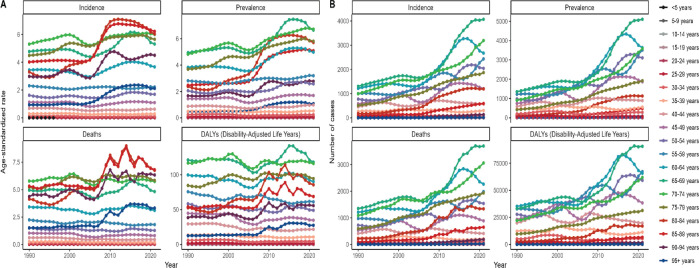
The graphs show that the ASIR, ASPR, ASDR and ASDAR of A-LC patients in China from 1990 to 2021 varied greatly among their respective age groups, while the differences among groups of disease-related cases were relatively insignificant.

Looking at the clustered lines on the right side of the figure, it can be seen that the light blue, dark green and light green coloured lines occupy the uppermost area of the graphs of the four epidemiological indicators for almost the entire period and have been increasing significantly since 2005; on the contrary, the colour lines of the age groups less than 40 years old have either been increasing slowly or have remained virtually unchanged (Figure 3b).

In other words, in terms of the number of liver cancer cases from 1990 to 2021, the number of alcohol-related primary liver cancer incidence, disease, death and disability-adjusted life-year cases in China is concentrated in the older age groups of 60–65, 66–69 and 70–74 years old, and the growth rate of the data generally culminated in a climax in the years of 2005–2021; while the number of liver cancer cases in people younger than 40 years old is lower. The number of liver cancer cases in the under age group was low and not significantly changing (Figure 3b).

Looking at the left graph, in addition to the light blue, dark green and light green lines similar to the right graph, three other colours, brown, dark red and light red, dominate the top of the graph, even beyond the blue–green series and show fluctuating increases and decreases in the later period (Figure 3a).

In other words, in terms of liver cancer-related ASRs from 1990 to 2021, A-LC ASIR, ASPR, ASDR and ASDAR in China were concentrated in the older age groups (60–65, 66–69, 70–74, 75–79, 80–84 and 85–89) in the 60–89 age group (Figure 3a), of which 80–84 was the oldest age group, Figure 3a), of which the ASDR and ASDAR of two age groups, 80–84 and 85–89 years old, showed three small peaks of values in the 3 years of 2010, 2014 and 2017, and simultaneously reached the maximum value of more than 7.5 and more than 100, respectively, in 2014 (Figure 3a).

The graph of the age group 60–64 years (light blue line), which was ranked second overall in the number of cases of A-LC incidence, prevalence, death and disability-adjusted life-years (ASDR, ASDAR), showed a similar trend from 1990 to 2021, i.e., the number of cases of A-LC firstly increased year by year and reached the maximum in 2016 or 2017, and then the number of prevalence gradually declined with the increase of time, and by 2021, the number of cases of A-LC The number of deaths declined to 2,266 (95% UI, 1,358–3,453) and the number of DALYs declined to 65,878 (95% UI, 39,452–100,372) (Figure 3b, Tables 3 and 4).

**Figure 4.**
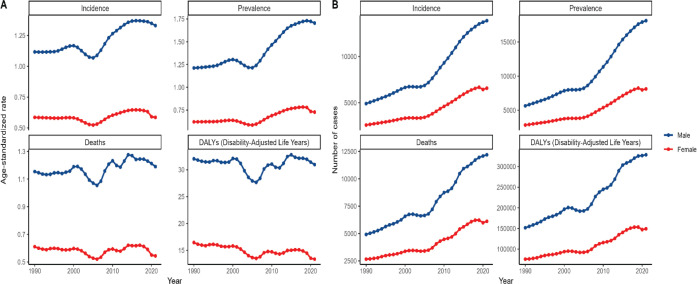
Depicts the trends of ASRs and corresponding number of cases associated with incidence, prevalence, death and disease-disability-adjusted life expectancy of A-LC patients of different genders in China from 1990 to 2021.

From 1990 to 2021, although the overall trends of ASIR, ASPR, ASDR, ASDAR and the corresponding number of cases of the indicators in Chinese patients with A-LC and HCC were similar between males and females under the same indicators, there were obvious gender differences in the values, which were manifested in the fact that males' values were constantly higher than those of females (Figure 4).

Interestingly, similar to the phenomenon of sudden trough (a sudden and accidental drop in the overall upward trend) that occurred in Figure 3, the number of cases of the four major indicators on the right side of Figure 4 also suddenly and significantly dropped to reach the bottom of the overall upward trend around 2005, and then rose or fluctuated upward (Figures 3b and 4b).

A closer look at the lines of male patients with a high incidence of liver cancer revealed that - first, the overall ASIR and ASPR of male liver cancer patients followed the same age pattern, with a small increase over time from 1990 to 2000, followed by a decline from 2000 to 2005, reaching a trough value in 2005, and then growing growing yearly until 2019 when it declined again slightly (Figure 4a).

Second, male ASIR reached a maximum value of over 1.25 in 2017, ASPR reached a maximum value close to 1.75 in 2019 and ASDR reached a maximum value close to 1.3 in 2014 (Figure 4a).

Third, the overall ASDR and ASDAR data for male liver cancer patients followed the same age pattern, with three distinct peaks and two distinct troughs in the graph lines, with the trough values occurring in 2006 and 2012, respectively (Figure 4a).

In terms of the overall trend of the ASR data from 1990 to 2021, the ASIR and ASDR values for male liver cancer patients were greater than 1, whereas the ASIR and ASDR values for females were less than 0.7; in terms of the ASDAR, it fluctuated above a constant 25 for males, whereas it was below a constant 18 for females and generally showed a decreasing trend over time (Figure 4a).

**Figure 5.**
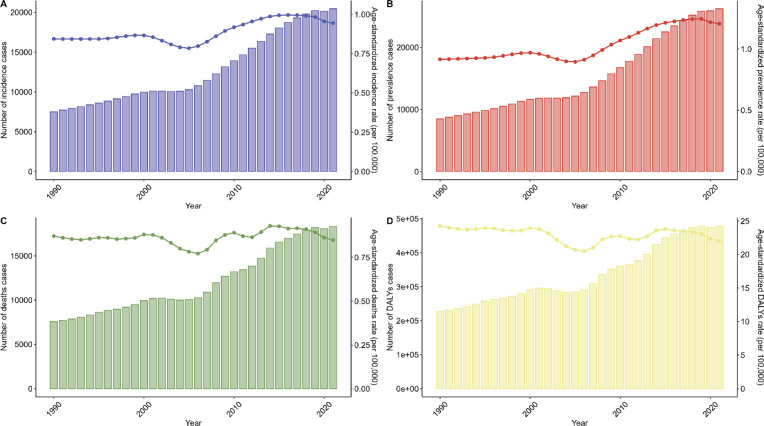
Shows the trends and numerical comparisons of the relevant age-standardised rates (per 100,000 population) and the respective corresponding number of cases for incidence, prevalence, death and DALYs of patients with alcohol-related liver cancer from 1990 to 2021.

From 1990 to 2021, the number of liver cancer patients with incidence and disease and the ASIR and ASPR, followed the same time-varying pattern, whereas the number of cases of disease and the corresponding change in ASR for the same metrics showed significant differences. During this period, the number of cases of A-LC patients in China generally grew over time, as shown by a small increase in the above four indicators from 1990 to 2000, a general plateau from 2000 to 2005, and a large increase in the number of cases from 2005 to 2021 (Figure 5a,b). The number of patient incidence cases, prevalence cases, and deaths increased to historical highs of 20,464 (95% UI, 15,239–27,296), 26,242 (95% UI, 19,417–34,985) and 18,317 (95% UI, 13,653–24,252) people, respectively (Figure 5, Table 1 incidence, Tables 2 and 3). EAPC (95% CI) were 0.62 (0.43–0.81), 1.15 (0.93–1.37) and 0.16 (0–0.32), respectively (Tables 1–3). The number of deaths and DALYs generally showed an upward trend and the fluctuating changes in ASDR and ASDAR all showed trough values in 2005 and 2012 (Figure 5c,d).

### Composition ratio of the three risk factors in A-LC, 1990 versus 2021

**Figure 6.**
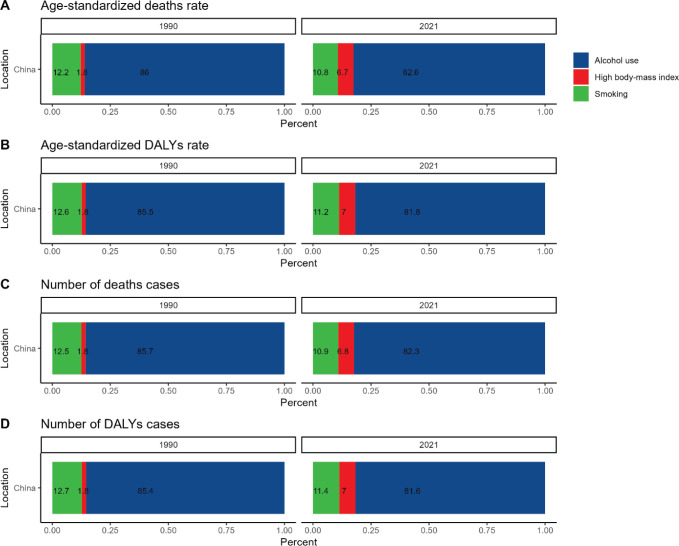
Analysis of A-LC-related risk factors.

Analysing Chinese liver cancer patients, we found the following among the three major risk factors for A-LC in China, alcohol abuse dominates with more than 80% share, smoking ranks second with more than 10% share and high body mass index (obesity H-BMI) ranks last with less than 10% share. However, despite high body mass index (obesity H-BMI) having the smallest share of the three major risk factors, obesity was the indicator that grew most significantly in 2021 compared with 1990; in contrast, alcohol abuse and cigarette smoking instead declined in their share of the three major risk factors for patients with HCC in 2021 (Figure 6).

Age-standardised mortality and DALYs caused by obesity increased from 1.8% to 6.7% and from 1.8% to 7%, respectively (Figure 6a,b) and age-standardised mortality and DALYs caused by alcoholism decreased from 86% to 82.6% and 85.5% to 81.8%, respectively (Figure 6a,b).

The proportion of patient deaths caused by obesity or alcohol abuse grew from 1.8% to 6.8% and 85.7% to 82.3%, respectively (Figure 6c). The percentage of DALYs for patients caused by obesity or alcohol abuse grew from 1.8% to 7% and decreased from 85.4% to 81.6%, respectively (Figure 6d).

The reason for this may be that since the 21st century, with the rapid change of scientific and technological progress and medical technology, the improvement of China's comprehensive national power and economic level, the increase of people's general life well-being index and the rise of health consciousness, the obese population in China has grown, while the population of alcoholism and smoking has greatly decreased and the proportion of liver cancer patients with obesity as a risk factor has grown, while the proportion of patients with traditional alcoholism and smoking has gradually declined. The proportion of liver cancer patients with obesity as a risk factor has increased significantly, while the proportion of traditional alcohol and smoking patients in liver cancer patients has gradually decreased.

### Two major models project A-LC trends from 2022 to 2050

**Figure 7.**
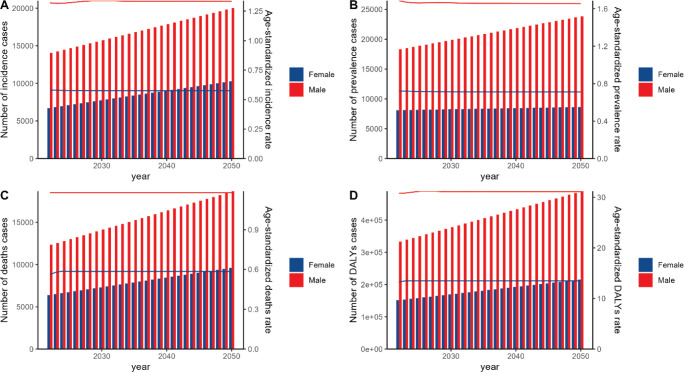
The predicted results in the A-LC-related numbers and age-standardised rates of ASIR, ASPR, ASDR and ASR of DALYs by sex in China from 2022 to 2050 of the ARIMA model.

**Figure 8.**
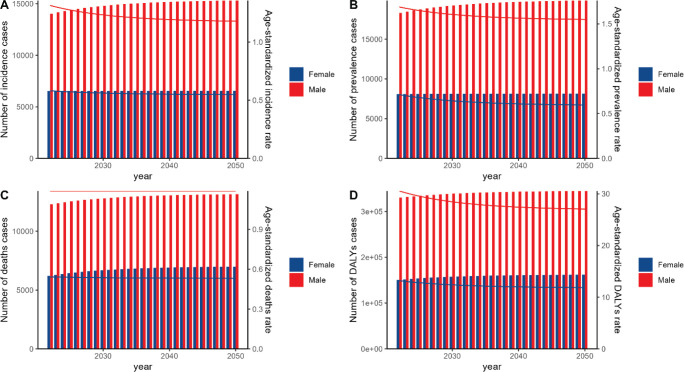
The predicted results in the A-LC-related numbers and age-standardised rates of ASIR, ASPR, ASDR and ASR of DALYs by sex in China from 2022 to 2050 of the ES model.

Predictions from the ARIMA and ES models suggest that between 2022 and 2050, the number of A-LC incidence cases, prevalence cases, deaths and DALYs will rise for both sexes. However, ASRs of incidence, prevalence, mortality and DALY are projected to decline for both sexes over this period (Figures 7 and 8; Tables 5 and 6).

## Discussion

In this study, we dug deeper into the GBD database for the years 1990–2021, comparing the GBD data reporting studies published in The Lancet for 371 diseases and injuries for 204 countries and territories and 811 subnational localities for the years 1990–2021 [14] explored the incidence, prevalence, mortality and DALYs for cancers in China during the years 1990–2021, corresponding to incidence, prevalence and the number of cancer patients, respectively. In 2021, China is projected to experience 20,464 cases of incidence, 26,242 cases of disease, 18,317 deaths and 477,847 DALYs. These figures have steadily increased from 1990 to 2021. During this period, the ASRs for morbidity and prevalence tended to increase, whereas the ASRs for mortality and DALYs tended to decrease. Our findings align with previous literature that the temporal trends in incidence rates vary considerably across regions and countries and are broadly consistent with regional trends in liver cancer mortality [15]. Previously reported trends of increasing incidence and deaths from primary liver cancer may be largely due to global population aging and population growth [8, 16–18]. In contrast to studies assessing the NAFLD-attributable burden of HCC, which report global increases in absolute deaths and ASDRs over the past decade, our findings highlight significant heterogeneity across different demographic groups.

We found that the main risk factor for A-LC cases and deaths in China from 1990 to 2021 was alcohol abuse, followed by smoking, obesity and other factors. Interestingly, the largest percentage increase in primary liver cancer risk factors for cases and deaths in China from 1990 to 2021 was obesity, in contrast to alcohol consumption, which accounted for more than 80% of cases in the past and smoking, which accounted for more than 10% of cases in the past, both of which accounted for varying degrees of reduction in 2021. In addition, the overall ASR of A-LC patients in China increased over time during 1990–2021, but the increasing trend was not consistent and the rate of increase varied greatly from year to year; and there was a nonlinear correlation between the four major ASR values of A-LC burden in 2021. Observation of our study shows that the ASR or the number of cases has increased considerably since 2005, which may be attributed to the fact that the incidence of hepatitis B-associated HCC has declined due to the widespread use of the hepatitis B vaccine globally in recent years [7, 19], but the incidence of hepatitis C-associated HCC has been growing [20, 21]. In other words, hepatitis-associated liver cancer was briefly prevalent after the vigorous development of the hepatitis B vaccine for primary liver cancer contributed to the widespread vaccination of the population. It is also interesting to note that it has been reported in the literature that the regional increase in ASDR for Liver cancer due to hepatitis C (LCDHC) grows with the increase in Socio-demographic Index (SDI), whereas there is no correlation between ASDR and SDI at the national and regional levels [22]. Despite the greatest increase in the percentage change in primary liver cancer cases and deaths between 1990 and 2019 in regions with high SDI, there was an increasing trend in ASIR and a stable trend in ASMR for primary liver cancer. The largest increase in ASIR was found in Oceania and the largest increase in ASMR was found in Central Asia [12]. Our study also found that the number of incident cases caused by A-LC grew and the ASDR of A-LC declined from 1990 to 2021, which is consistent with the findings of a previous study that reported that the ASDR of HCC declined globally [23].

Previous studies have reported that hepatitis B and C are the main causes of liver cancer and alcohol abuse is the most important risk factor for liver cancer in China [24, 25]. In 2021, alcohol abuse, a risk factor, accounted for approximately 82.3% of liver cancer deaths and obesity accounted for approximately 6.8%; compared to 1919, when the proportion of liver cancer deaths contributed to by obesity factors was only 1.8% and the increase in the obese population is a global trend [26]. In recent years, due to the successful development of the Healthy China strategy and the general improvement of Chinese people's living standards, the incidence of alcoholism-associated liver cancer has declined, but the incidence of obesity-induced-associated liver cancer has been growing. The disease burden of A-LC induced by alcoholism and smoking as the traditional percentage has been decreasing, while the disease burden of obesity-induced A-LC has been increasing rapidly between 1990 and 2021. Thus, obesity has become one of the fastest-rising causes of liver cancer share. In addition to addressing alcohol abuse, understanding the burden of obesity-induced HCC may help to reduce the overall burden of the disease [27, 28].

In our study, we found a high number of incidence cases in the age group of 50–89 years, suggesting that the trend of older age groups accounting for the majority of patients with A-LC is in line with previous reports in the literature [12, 13, 23]. Tables 1 and 3 show that in 2021, the burden of liver cancer in the age group of 50 years and above will be almost more than 90% of the total prevalence (89.06% incidence and 91.11% mortality). Findings from numerous previous studies recommend screening for HCV in older adults to facilitate early detection of infection and timely initiation of treatment. HCV-infected older adults should receive prompt and effective treatment to reduce the risk of liver cancer [22, 29]. In addition, people over 50 years of age and elderly HCV patients should undergo routine monitoring of liver function and early detection of any signs of liver cancer for prevention [30]. The ASIR of LCDHC has also been reported to show the greatest increase in Asia-Pacific, North Africa, the Middle East and Central Asia from 1990 to 2019 and HCV is the leading cause of primary liver cancer in high SDI high regions, with HCC caused by chronic hepatitis C infections accounting for 43% and 41.4% of the deaths and morbidity cases of HCC, respectively, in these regions [31]. It has been documented that certain high-income regions, such as Japan, have a higher ASIR for LCDHC than most countries. Despite having more resources, including financial resources, healthcare infrastructure and medical technology, high-income countries are not immune to the burden of HCV infection and LCDHC [29]. Therefore, the development of an effective HCV vaccine is essential to achieve the goal of eliminating HCV by 2030 [32].

In addition to differences in different age groups, our study also found a significant difference in the burden of A-LC between the sexes.2021 The highest incidence of A-LC in the population suffering from liver cancer was found in alcoholic obese older men. The incidence of A-LC was generally higher in males than in females, a trend that has persisted across all stages of the age group, a trend that is similar to the findings of previous studies in the literature [13]. The reasons for this higher rate in males than females may be as follows: males are more likely to be alcoholics, smokers and more likely to be obese in stature. Hormonal differences between males and females may also play a role, as oestrogen has been shown to have a protective effect against liver cancer and oestrogen levels are usually higher in females than in males [33]. In addition, it has been suggested that testosterone may promote the growth of HCC cells, which may also contribute to the gender difference in the incidence of HCV-associated HCC [34].

To date, there are no clinical treatments that can effectively cure patients in complete remission of A-LC patients. Therefore, strengthening the control of alcohol intake in male at-risk groups and urging obese HBMI-statured at-risk groups to lose weight and become fit are the main strategies to reduce the burden of A-LC. Due to the lack of an effective vaccine to prevent HCV infection, despite the introduction of targeted antiviral drug regimens a decade ago, which can cure more than 90% of HCV patients, high costs, drug resistance and reinfection rates remain significant barriers to achieving this goal [35, 36]. Despite advancements in global healthcare standards and increased awareness of HCV, the detection rate of HCC remains suboptimal. This has contributed to a decline in ASIRs. However, many HCV patients lack access to effective treatment and subsequently develop HCC [32]. Therefore, evaluating the effectiveness of prevention programs, comparing observed values and expected levels in each country and region while considering other factors (e.g., the prevalence of hepatitis C infection, availability of healthcare and screening programs, availability of effective treatment programs, demographic factors) and then comprehensively developing and designing an effective clinical treatment process for patients with A-LC is essential to achieve a sustained reduction in the burden of HCC and even the goal of eliminating A-LC by 2050 is critical.

The main strengths of this study include the analysis of the most recent data on the A-LC burden in China, specifically focusing on ASRs for mortality, DALYs, incidence and prevalence related to the major causes of A-LC. To our knowledge, this is the latest comprehensive examination of the A-LC burden in China, along with predictions of trends over the next 25 years, including associated risk factors.

However, there are limitations to consider. First, data quality and completeness: Our estimates for A-LC burden in China are based on the raw data underlying GBD 2021. In the 1990s, disease registries were less developed and internet access was limited, which likely led to incomplete registrations and possible underestimation of true case counts. Modeling with incomplete data: In regions or periods with sparse data, estimates rely on statistical modeling and historical trends. This can introduce uncertainty, variability and potential discrepancies across regions and over time. Second, regional granularity: While we present subnational patterns, the analysis does not capture fine-grained country-specific details that may reflect important local variations in risk factors, healthcare access and reporting practices within China. In addition, epidemiologic data can be inconsistent, as the diagnostic and surveillance systems are constantly changing. Finally, Lack of genetic information: The GBD database does not include genetic data, which limits our ability to explore the joint influence of environmental exposures and genetic susceptibility on disease burden. Potential confounding and attribution: Attribution of liver cancer burden to alcohol may be affected by coexisting risk factors (e.g., hepatitis B/C infections, aflatoxin exposure, obesity). Regional differences in these factors could bias the estimated alcohol-attributable burden. Changes in definitions and coding: Shifts in disease definitions, coding practices or diagnostic criteria over the study period may affect comparability across years. Generalizability: Although the findings are robust at national and regional levels for China, caution is needed when extrapolating to other populations with different risk profiles and health systems. Despite these limitations, the analysis provides valuable, policy-relevant insights into long-term trends and regional patterns of A-LC burden in China.

## Conclusion

Although the ASR associated with A-LC has declined in the last 3 years, the absolute numbers of morbidity cases, mortality and DALYs have increased, suggesting that A-LC remains a significant burden, at least in China. From the perspective of gender, there is a substantial difference in the risk of ALC between men and women and the ASR associated with morbidity and mortality is always significantly higher in men than in women. The ARIMA model predicts that by 2050, the incidence of male A-LC patients in China will exceed 20,000 the number of deaths will continue to increase by nearly 20,000 and the overall ASR of men will remain more than twice the level of women. Our findings provide insights into trends in China's A-LC burden in 2050 and can help policymakers develop appropriate approaches to reduce the disease burden of A-LC in China ultimately. Focusing on these burden trends is critical, especially as the number of A-LC cases and the aging population continue to grow.

## Conflicts of interest

We declare that we have no financial and personal relationships with other people or organizations that can inappropriately influence our work, there is no professional or other personal interest of any nature or kind in any product, service and company that could be construed as influencing the position presented in or the review of the manuscript entitled.

## Author contributions

Managing the overall research enterprise: Nan Luo and Pengkhun Nov; Writing the first draft of the manuscript: Pengkhun Nov, Juanli Xu and Jiqiang Li; Primary responsibility for applying analytical methods to produce estimates: Nan Luo; Primary responsibility for seeking, cataloguing, extracting or cleaning data; designing or coding figures and tables, providing data or critical feedback on data sources: Prasai Arzoo.

## Data availability

This study follows the Guidelines for Accurate and Transparent Health Estimates Reporting (GATHER). To download the data used in these analyses, please visit the Global Health Data Exchange (GHDx). https://ghdx.healthdata.org/gbd-2021.

## Ethical approval

Not applicable because this study extracted data from database.

## Figures and Tables

**Table 1.** The number of incidence cases and the ASIR attributable to A-LC in 1990 and 2021, and its trends from 1990 to 2021 in China.

Characteristics	Number of incidence cases (95% UI) in 1990	The ASIR/100,000 (95% UI) in 1990	Number of incidence cases (95% UI) in 2021	The ASIR/100,000 (95% UI) in 2021	EAPC (95% CI)
China	7,500 (5,772–9,563)	0.84 (0.66–1.07)	20,464 (15,239–27,296)	0.94 (0.71–1.25)	0.62 (0.43–0.81)
Sex					
Female	2,591 (1,924–3,345)	0.59 (0.43–0.75)	6,579 (4,820–8,697)	0.58 (0.43–0.77)	0.32 (0.12–0.52)
Male	4,909 (3,692–6,547)	1.12 (0.84–1.45)	13,885 (9,569–19,671)	1.33 (0.93–1.85)	0.81 (0.62–0.99)
Age					
15–19 years	2 (1–3)	0 (0–0)	1 (0–1)	0 (0–0)	−1.35 (−1.58 to −1.11)
20–24 years	10 (7–15)	0.01 (0.01–0.01)	5 (3–7)	0.01 (0–0.01)	−2 (−2.47 to −1.54)
25–29 years	34 (20–54)	0.03 (0.02–0.05)	26 (16–42)	0.03 (0.02–0.05)	−0.97 (−1.69 to −0.24)
30–34 years	98 (67–136)	0.11 (0.08–0.15)	124 (83–185)	0.1 (0.07–0.15)	−1.05 (−1.6 to −0.49)
35–39 years	267 (167–397)	0.29 (0.18–0.43)	303 (180–458)	0.29 (0.17–0.43)	−0.63 (−0.91 to −0.36)
40–44 years	456 (297–663)	0.68 (0.44–0.99)	571 (354–813)	0.62 (0.39–0.89)	−0.69 (−1.01 to −0.37)
45–49 years	580 (353–884)	1.12 (0.68–1.71)	1,207 (729–1,886)	1.09 (0.66–1.71)	−0.01 (−0.43 to 0.41)
50–54 years	775 (495–1,163)	1.62 (1.04–2.44)	2,044 (1,256–3,120)	1.69 (1.04–2.58)	0.56 (0.31–0.81)
55–59 years	1,001 (620–1,536)	2.31 (1.43–3.54)	2,437 (1,370–3,927)	2.22 (1.25–3.57)	−0.05 (−0.16 to 0.06)
60–64 years	1,215 (781–1,826)	3.44 (2.21–5.17)	2,674 (1,598–4,092)	3.66 (2.19–5.61)	0.56 (0.26–0.85)
65–69 years	1,301 (911–1,821)	4.77 (3.34–6.67)	4,072 (2,796–5,762)	5.31 (3.64–7.51)	0.72 (0.42–1.02)
70–74 years	997 (702–1,383)	5.3 (3.73–7.35)	3,197 (2,231–4,647)	6 (4.19–8.72)	0.32 (0.18–0.47)
75–79 years	511 (379–660)	4.49 (3.33–5.8)	1,870 (1,362–2,555)	5.65 (4.11–7.71)	0.94 (0.8–1.07)
80–84 years	175 (128–233)	3.3 (2.41–4.4)	1,194 (841–1,624)	6.03 (4.25–8.21)	3.44 (2.92–3.96)
85–89 years	68 (51–88)	4.02 (3.02–5.22)	592 (439–787)	6.22 (4.61–8.27)	2.19 (1.77–2.61)
90–94 years	9 (7–12)	2.98 (2.16–4.06)	132 (93–182)	4.5 (3.18–6.22)	1.73 (1.35–2.1)
95+ years	0 (0–1)	0.93 (0.57–1.44)	14 (8–21)	2.11 (1.23–3.35)	3.96 (3.37–4.55)

**Table 2.** The number of prevalence cases and the ASPR attributable to A-LC in 1990 and 2021, and its trends from 1990 to 2021 in China.

Characteristics	Number of prevalence cases (95% UI) in 1990	The ASPR/100,000 (95% UI) in 1990	Number of prevalence cases (95% UI) in 2021	The ASPR/100,000 (95% UI) in 2021	EAPC (95% CI)
China	8,484 (6,509–10,955)	0.92 (0.71–1.17)	26,242 (19,417–34,985)	1.21 (0.9–1.59)	1.15 (0.93–1.37)
Sex					
Female	2,837 (2,094–3,704)	0.62 (0.46–0.81)	8,125 (5,909–10,777)	0.73 (0.53–0.96)	0.83 (0.62–1.05)
Male	5,648 (4,255–7,527)	1.21 (0.92–1.6)	18,117 (12,306–25,853)	1.7 (1.17–2.41)	1.35 (1.13–1.57)
Age					
15–19 years	3 (1–5)	0 (0–0)	1 (1–3)	0 (0–0)	−1.02 (−1.24 to −0.8)
20–24 years	17 (11–24)	0.01 (0.01–0.02)	8 (5–13)	0.01 (0.01–0.02)	−1.69 (−2.15 to −1.21)
25–29 years	79 (47–124)	0.07 (0.04–0.11)	67 (39–105)	0.08 (0.05–0.12)	−0.66 (−1.39 to 0.08)
30–34 years	205 (142–285)	0.23 (0.16–0.32)	282 (189–420)	0.23 (0.16–0.35)	−0.79 (−1.35 to −0.22)
35–39 years	449 (280–667)	0.49 (0.31–0.73)	555 (330–827)	0.52 (0.31–0.78)	−0.34 (−0.63 to −0.06)
40–44 years	587 (382–853)	0.87 (0.57–1.27)	893 (556–1,264)	0.98 (0.61–1.38)	−0.01 (−0.34 to 0.33)
45–49 years	746 (454–1,133)	1.45 (0.88–2.2)	1,869 (1,132–2,916)	1.69 (1.03–2.64)	0.63 (0.21–1.05)
50–54 years	980 (625–1,471)	2.05 (1.31–3.08)	3,108 (1,920–4,754)	2.57 (1.59–3.93)	1.22 (0.95–1.48)
55–59 years	1,219 (755–1,870)	2.81 (1.74–4.31)	3,512 (1,975–5,623)	3.19 (1.8–5.11)	0.56 (0.44–0.69)
60–64 years	1,352 (869–2,029)	3.83 (2.46–5.74)	3,632 (2,162–5,542)	4.98 (2.96–7.59)	1.26 (0.94–1.58)
65–69 years	1,348 (942–1,883)	4.94 (3.45–6.9)	5,104 (3,499–7,246)	6.65 (4.56–9.45)	1.38 (1.06–1.71)
70–74 years	907 (637–1,261)	4.82 (3.39–6.7)	3,583 (2,497–5,243)	6.72 (4.68–9.84)	1.06 (0.9–1.22)
75–79 years	420 (311–542)	3.69 (2.74–4.76)	1,923 (1,392–2,640)	5.81 (4.2–7.97)	1.72 (1.6–1.84)
80–84 years	126 (92–168)	2.38 (1.74–3.17)	1,131 (793–1,539)	5.72 (4–7.78)	4.46 (3.93–5)
85–89 years	42 (31–54)	2.47 (1.86–3.21)	484 (356–641)	5.09 (3.74–6.73)	3.18 (2.74–3.63)
90–94 years	5 (4–7)	1.67 (1.21–2.28)	81 (57–112)	2.77 (1.96–3.82)	2.05 (1.68–2.43)
95+ years	0 (0–0)	0.47 (0.29–0.73)	7 (4–11)	1.07 (0.62–1.7)	3.98 (3.39–4.57)

**Table 3.** The number of deaths cases and the ASDR attributable to A-LC in 1990 and 2021, and its trends from 1990 to 2021 in China.

Characteristics	Number of deaths cases (95% UI) in 1990	The ASDR/100,000 (95% UI) in 1990	Number of deaths cases (95% UI) in 2021	The ASDR/100,000 (95% UI) in 2021	EAPC (95% CI)
China	7,575 (5,858–9,677)	0.87 (0.69–1.1)	18,317 (13,653–24,252)	0.85 (0.64–1.12)	0.16 (0−0.32)
Sex					
Female	2,659 (1,976–3,433)	0.61 (0.46–0.78)	6,119 (4,472–8,144)	0.54 (0.4–0.73)	−0.04 (−0.22 to 0.14)
Male	4,915 (3,680–6,553)	1.15 (0.87–1.49)	12,198 (8,489–17,015)	1.19 (0.85–1.62)	0.31 (0.16–0.46)
Age					
15–19 years	2 (1–3)	0 (0–0)	1 (0–1)	0 (0–0)	−2.08 (−2.4 to −1.76)
20–24 years	11 (7–15)	0.01 (0.01–0.01)	4 (2–6)	0.01 (0–0.01)	−2.61 (−3.1 to −2.12)
25–29 years	32 (19–49)	0.03 (0.02–0.05)	20 (12–31)	0.02 (0.01–0.04)	−1.65 (−2.41 to −0.89)
30–34 years	89 (61–124)	0.1 (0.07–0.14)	92 (62–137)	0.08 (0.05–0.11)	−1.74 (−2.33 to −1.14)
35–39 years	231 (144–342)	0.25 (0.16–0.37)	213 (127–317)	0.2 (0.12–0.3)	−1.28 (−1.55 to −1.01)
40–44 years	411 (267–601)	0.61 (0.4–0.9)	410 (254–591)	0.45 (0.28–0.65)	−1.48 (−1.76 to −1.2)
45–49 years	535 (323–814)	1.04 (0.63–1.58)	889 (532–1,389)	0.81 (0.48–1.26)	−0.82 (−1.24 to −0.4)
50–54 years	725 (463–1,100)	1.52 (0.97–2.31)	1,540 (941–2,358)	1.27 (0.78–1.95)	−0.29 (−0.5 to −0.09)
55–59 years	972 (601–1,508)	2.24 (1.39–3.48)	1,988 (1,133–3,194)	1.81 (1.03–2.9)	−0.61 (−0.76 to −0.46)
60–64 years	1,211 (776–1,828)	3.43 (2.2–5.17)	2,266 (1,358–3,453)	3.1 (1.86–4.73)	−0.02 (−0.25 to 0.22)
65–69 years	1,351 (948–1,885)	4.95 (3.47–6.91)	3,700 (2,546–5,230)	4.82 (3.32–6.82)	0.23 (−0.05 to 0.5)
70–74 years	1,090 (767–1,518)	5.79 (4.07–8.07)	3,062 (2,139–4,470)	5.75 (4.01–8.39)	−0.11 (−0.25 to 0.04)
75–79 years	594 (441–766)	5.22 (3.88–6.73)	1,937 (1,413–2,646)	5.85 (4.27–7.99)	0.58 (0.42–0.75)
80–84 years	219 (160–293)	4.14 (3.03–5.54)	1,339 (941–1,799)	6.76 (4.75–9.09)	2.96 (2.46–3.45)
85–89 years	90 (67–116)	5.31 (4–6.9)	649 (481–859)	6.82 (5.05–9.02)	1.56 (1.15–1.97)
90–94 years	13 (10–18)	4.37 (3.17–5.95)	186 (132–257)	6.34 (4.49–8.76)	1.49 (1.11–1.88)
95+ years	1 (0–1)	1.5 (0.91–2.32)	21 (12–33)	3.32 (1.95–5.23)	3.58 (3.02–4.15)

**Table 4.** The number of DALYs cases and the age-standardised DALYs rate attributable to A-LC in 1990 and 2021, and its trends from 1990 to 2021 in China.

Characteristics	Number of DALYs cases (95% UI) in 1990	The age-standardised DALYs rate/100,000 (95% UI) in 1990	Number of DALYs cases (95% UI) in 2021	The age-standardised DALYs rate/100,000 (95% UI) in 2021	EAPC (95% CI)
China	227,509 (174,534–293,034)	24.26 (18.72–31.11)	477,847 (352,518–637,755)	22.01 (16.3–29.15)	−0.18 (−0.34 to −0.01)
Sex					
Female	75,746 (55,929–98,525)	16.45 (12.21–21.46)	149,332 (107,233–198,685)	13.34 (9.6–17.61)	–0.46 (−0.61 to −0.3)
Male	151,763 (113,195–203,754)	32.04 (24.17–42.63)	328,515 (220,222–469,326)	30.96 (21.08–43.77)	0 (−0.16 to 0.17)
Age					
15–19 years	124 (54–216)	0.1 (0.04–0.17)	44 (21–80)	0.06 (0.03–0.11)	−2.08 (−2.41 to −1.76)
20–24 years	714 (480–1,011)	0.54 (0.36–0.77)	262 (160–405)	0.36 (0.22–0.55)	−2.62 (−3.1 to −2.12)
25–29 years	1,991 (1,186–3,126)	1.81 (1.08–2.85)	1,238 (732–1,965)	1.43 (0.85–2.27)	−1.66 (−2.41 to −0.89)
30–34 years	5,139 (3,546–7,144)	5.82 (4.02–8.1)	5,333 (3,577–7,906)	4.4 (2.95–6.53)	−1.73 (−2.33 to −1.13)
35–39 years	12,265 (7,643–18,183)	13.43 (8.37–19.91)	11,337 (6,753–16,861)	10.7 (6.37–15.91)	−1.28 (−1.55 to −1)
40–44 years	19,780 (12,846–28,907)	29.48 (19.15–43.08)	19,736 (12,255–28,445)	21.56 (13.39–31.08)	−1.49 (−1.77 to −1.21)
45–49 years	23,097 (13,957–35,188)	44.75 (27.04–68.17)	38,368 (22,945–59,870)	34.78 (20.8–54.27)	−0.82 (−1.24 to −0.4)
50–54 years	27,815 (17,723–42,175)	58.3 (37.15–88.4)	59,179 (36,251–90,622)	48.97 (29.99–74.98)	−0.28 (−0.49 to −0.07)
55–59 years	32,812 (20,313–50,836)	75.66 (46.84–117.22)	67,289 (38,286–108,111)	61.2 (34.82–98.33)	−0.61 (−0.76 to −0.45)
60–64 years	35,245 (22,593–53,210)	99.74 (63.93–150.58)	65,878 (39,452–100,372)	90.24 (54.04–137.49)	−0.02 (−0.26 to 0.22)
65–69 years	33,092 (23,218–46,264)	121.3 (85.11–169.58)	90,654 (62,375–128,156)	118.19 (81.32–167.08)	0.24 (−0.04 to 0.51)
70–74 years	21,987 (15,501–30,661)	116.84 (82.37–162.94)	61,912 (43,342–90,471)	116.17 (81.32–169.75)	−0.11 (−0.25 to 0.04)
75–79 years	9,630 (7,172–12,404)	84.61 (63.02–108.99)	31,321 (22,783–42,800)	94.57 (68.79–129.23)	0.57 (0.4–0.73)
80–84 years	2,787 (2,040–3,729)	52.61 (38.51–70.4)	16,932 (11,932–22,763)	85.55 (60.29–115.01)	2.94 (2.45–3.43)
85–89 years	909 (685–1,180)	53.89 (40.58–69.93)	6,556 (4,875–8,675)	68.83 (51.18–91.07)	1.56 (1.15–1.97)
90–94 years	118 (86–161)	38.46 (27.9–52.46)	1,632 (1,153–2,252)	55.67 (39.32–76.82)	1.49 (1.11–1.88)
95+ years	5 (3–8)	12.52 (7.67–19.4)	176 (103–278)	27.51 (16.16–43.43)	3.57 (2.99–4.14)

**Table 5.** The number of predict cases of A–LC in China from 2022 to 2050 by ARIMA model.

Year	Sex	Age–standardised incidence rate	Numer of incidence cases	ASPR	Numer of prevalence cases	ASDR	Numer of deaths cases	Age–standardised DALYs rate	Numer of DALYs cases
2022	Male	1.319719862	14064.4105	1.685496569	18314.35322	1.179675278	12354.00778	30.78158232	333209.8474
2023	Male	1.315603787	14261.87883	1.670855229	18511.83222	1.179675278	12553.47239	30.84264838	339234.1955
2024	Male	1.316733887	14471.07062	1.663076641	18709.31123	1.179675278	12780.61787	31.00624358	344812.29
2025	Male	1.321058202	14685.46569	1.661112384	18906.79023	1.179675278	13007.76334	31.1465683	350390.3846
2026	Male	1.326566659	14901.03704	1.662354073	19104.26923	1.179675278	13234.90882	31.20672022	355968.4792
2027	Male	1.331697084	15116.13024	1.664209509	19301.74823	1.179675278	13462.0543	31.19520686	361546.5738
2028	Male	1.335507011	15330.45858	1.665047278	19499.22723	1.179675278	13689.19978	31.15169975	367124.6684
2029	Male	1.33765638	15544.2511	1.664402954	19696.70624	1.179675278	13916.34525	31.11314632	372702.7629
2030	Male	1.338270208	15757.79358	1.662663977	19894.18524	1.179675278	14143.49073	31.09683009	378280.8575
2031	Male	1.337751486	15971.27149	1.660561465	20091.66424	1.179675278	14370.63621	31.10094245	383858.9521
2032	Male	1.336600671	16184.76528	1.658738941	20289.14324	1.179675278	14597.78168	31.11431384	389437.0467
2033	Male	1.335277265	16398.29191	1.657530406	20486.62224	1.179675278	14824.92716	31.12627546	395015.1413
2034	Male	1.334117854	16611.84289	1.656947519	20684.10125	1.179675278	15052.07264	31.13171996	400593.2359
2035	Male	1.333308235	16825.40581	1.65679634	20881.58025	1.179675278	15279.21811	31.13107543	406171.3304
2036	Male	1.332896729	17038.97215	1.656826865	21079.05925	1.179675278	15506.36359	31.12755213	411749.425
2037	Male	1.33283165	17252.53805	1.656845055	21276.53825	1.179675278	15733.50907	31.12428095	417327.5196
2038	Male	1.333006724	17466.10256	1.656759601	21474.01726	1.179675278	15960.65454	31.12280842	422905.6142
2039	Male	1.333302216	17679.66596	1.656571682	21671.49626	1.179675278	16187.80002	31.12306714	428483.7088
2040	Male	1.333614635	17893.2288	1.656334351	21868.97526	1.179675278	16414.9455	31.1241563	434061.8033
2041	Male	1.333872765	18106.79146	1.656109069	22066.45426	1.179675278	16642.09097	31.12517345	439639.8979
2042	Male	1.334041393	18320.35413	1.655936955	22263.93326	1.179675278	16869.23645	31.12566295	445217.9925
2043	Male	1.334116215	18533.91685	1.655829685	22461.41227	1.179675278	17096.38193	31.12563639	450796.0871
2044	Male	1.334114042	18747.47963	1.655775542	22658.89127	1.179675278	17323.5274	31.1253524	456374.1817
2045	Male	1.334061973	18961.04243	1.655752175	22856.37027	1.179675278	17550.67288	31.12507562	461952.2762
2046	Male	1.33398817	19174.60524	1.655738478	23053.84927	1.179675278	17777.81836	31.12494365	467530.3708
2047	Male	1.333915594	19388.16805	1.655721382	23251.32827	1.179675278	18004.96383	31.12495801	473108.4654
2048	Male	1.333859002	19601.73086	1.655697003	23448.80728	1.179675278	18232.10931	31.1250464	478686.56
2049	Male	1.333824703	19815.29367	1.655667944	23646.28628	1.179675278	18459.25479	31.12513269	484264.6546
2050	Male	1.333812157	20028.85648	1.655639301	23843.76528	1.179675278	18686.40026	31.12517644	489842.7491
2022	Female	0.581465552	6715.908531	0.722890564	8088.345517	0.562634981	6392.480897	13.25805112	151482.9218
2023	Female	0.579184852	6848.678024	0.719570012	8132.092734	0.58037491	6507.143285	13.4857023	153687.7325
2024	Female	0.577691235	6979.00535	0.717275433	8140.047754	0.587433857	6621.805673	13.4857023	155924.3344
2025	Female	0.576713074	7107.853562	0.715689824	8163.975526	0.584979143	6736.468061	13.4857023	158179.7047
2026	Female	0.576072481	7235.805939	0.714594131	8180.775241	0.584979143	6851.130449	13.4857023	160446.1552
2027	Female	0.575652961	7363.215746	0.713836981	8200.755949	0.584979143	6965.792837	13.4857023	162719.1469
2028	Female	0.57537822	7490.296943	0.713313773	8219.317095	0.584979143	7080.455225	13.4857023	164996.0005
2029	Female	0.575198294	7617.179115	0.712952223	8238.51174	0.584979143	7195.117613	13.4857023	167275.1339
2030	Female	0.575080461	7743.940746	0.712702384	8257.423677	0.584979143	7309.780001	13.4857023	169555.6132
2031	Female	0.575003293	7870.629371	0.712529739	8276.461777	0.584979143	7424.442389	13.4857023	171836.8871
2032	Female	0.574952756	7997.273779	0.712410438	8295.443575	0.584979143	7539.104777	13.4857023	174118.6302
2033	Female	0.57491966	8123.891406	0.712327998	8314.450498	0.584979143	7653.767165	13.4857023	176400.6501
2034	Female	0.574897985	8250.492814	0.712271029	8333.446209	0.584979143	7768.429553	13.4857023	178682.8336
2035	Female	0.574883791	8377.084399	0.712231663	8352.446924	0.584979143	7883.091941	13.4857023	180965.1136
2036	Female	0.574874495	8503.670034	0.71220446	8371.445405	0.584979143	7997.754329	13.4857023	183247.4505
2037	Female	0.574868407	8630.252065	0.712185662	8390.444883	0.584979143	8112.416716	13.4857023	185529.8211
2038	Female	0.57486442	8756.831914	0.712172672	8409.443917	0.584979143	8227.079104	13.4857023	187812.2116
2039	Female	0.574861809	8883.410441	0.712163696	8428.443148	0.584979143	8341.741492	13.4857023	190094.6138
2040	Female	0.574860099	9009.988167	0.712157493	8447.442292	0.584979143	8456.40388	13.4857023	192377.0229
2041	Female	0.574858979	9136.565409	0.712153207	8466.441474	0.584979143	8571.066268	13.4857023	194659.4361
2042	Female	0.574858246	9263.142357	0.712150245	8485.44064	0.584979143	8685.728656	13.4857023	196941.8517
2043	Female	0.574857766	9389.719127	0.712148198	8504.439813	0.584979143	8800.391044	13.4857023	199224.2688
2044	Female	0.574857451	9516.295789	0.712146784	8523.438982	0.584979143	8915.053432	13.4857023	201506.6867
2045	Female	0.574857245	9642.872386	0.712145806	8542.438153	0.584979143	9029.71582	13.4857023	203789.105
2046	Female	0.57485711	9769.448943	0.712145131	8561.437323	0.584979143	9144.378208	13.4857023	206071.5237
2047	Female	0.574857022	9896.025477	0.712144664	8580.436494	0.584979143	9259.040596	13.4857023	208353.9426
2048	Female	0.574856964	10022.602	0.712144342	8599.435665	0.584979143	9373.702984	13.4857023	210636.3615
2049	Female	0.574856926	10149.17851	0.712144119	8618.434835	0.584979143	9488.365372	13.4857023	212918.7805
2050	Female	0.574856901	10275.75501	0.712143965	8637.434006	0.584979143	9603.02776	13.4857023	215201.1995

**Table 6.** The number of predict cases of A–LC in China from 2022 to 2050 by ES model.

Year	Sex	Age–standardised incidence rate	Numer of incidence cases	ASPR	Numer of prevalence cases	ASDR	Numer of deaths cases	Age–standardised DALYs rate	Numer of DALYs cases
2022	Male	1.314689089	14034.50216	1.68860667	18300.41746	1.189650516	12297.72684	30.55256505	330212.7042
2023	Male	1.300421275	14169.41722	1.673976085	18465.60047	1.189635629	12387.30582	30.1850349	331740.755
2024	Male	1.287580243	14290.84078	1.660808559	18614.26518	1.18962223	12467.9269	29.85425776	333116.0008
2025	Male	1.276023314	14400.12198	1.648957786	18748.06342	1.189610171	12540.48587	29.55655834	334353.722
2026	Male	1.265622078	14498.47506	1.63829209	18868.48183	1.189599318	12605.78895	29.28862886	335467.671
2027	Male	1.256260965	14586.99283	1.628692963	18976.8584	1.18958955	12664.56172	29.04749233	336470.2252
2028	Male	1.247835964	14666.65883	1.620053749	19074.39732	1.189580759	12717.45721	28.83046945	337372.5239
2029	Male	1.240253463	14738.35823	1.612278457	19162.18234	1.189572847	12765.06315	28.63514886	338184.5928
2030	Male	1.233429212	14802.88768	1.605280694	19241.18886	1.189565726	12807.9085	28.45936032	338915.4548
2031	Male	1.227287386	14860.9642	1.598982707	19312.29473	1.189559318	12846.46931	28.30115065	339573.2305
2032	Male	1.221759742	14913.23306	1.593314518	19376.29001	1.18955355	12881.17404	28.15876193	340165.2287
2033	Male	1.216784863	14960.27503	1.588213149	19433.88577	1.189548359	12912.4083	28.03061209	340698.0271
2034	Male	1.212307472	15002.61281	1.583621917	19485.72195	1.189543687	12940.51913	27.91527724	341177.5457
2035	Male	1.20827782	15040.71681	1.579489807	19532.37451	1.189539482	12965.81888	27.81147587	341609.1124
2036	Male	1.204651133	15075.0104	1.575770909	19574.36181	1.189535698	12988.58866	27.71805463	341997.5224
2037	Male	1.201387115	15105.87464	1.572423901	19612.15039	1.189532292	13009.08145	27.63397553	342347.0914
2038	Male	1.198449499	15133.65246	1.569411593	19646.1601	1.189529227	13027.52497	27.55830433	342661.7035
2039	Male	1.195805644	15158.65249	1.566700516	19676.76885	1.189526468	13044.12414	27.49020025	342944.8544
2040	Male	1.193426175	15181.15252	1.564260547	19704.31672	1.189523986	13059.06339	27.42890658	343199.6902
2041	Male	1.191284653	15201.40255	1.562064575	19729.1098	1.189521751	13072.50871	27.37374227	343429.0425
2042	Male	1.189357283	15219.62758	1.5600882	19751.42358	1.18951974	13084.6095	27.3240944	343635.4595
2043	Male	1.18762265	15236.0301	1.558309462	19771.50598	1.18951793	13095.50021	27.27941131	343821.2348
2044	Male	1.18606148	15250.79237	1.556708598	19789.58013	1.189516301	13105.30185	27.23919653	343988.4326
2045	Male	1.184656427	15264.07841	1.555267821	19805.84688	1.189514835	13114.12333	27.20300323	344138.9106
2046	Male	1.18339188	15276.03585	1.553971121	19820.48694	1.189513515	13122.06266	27.17042926	344274.3408
2047	Male	1.182253787	15286.79754	1.552804092	19833.663	1.189512328	13129.20806	27.14111269	344396.2279
2048	Male	1.181229504	15296.48307	1.551753765	19845.52146	1.189511259	13135.63891	27.11472778	344505.9264
2049	Male	1.180307648	15305.20004	1.550808471	19856.19407	1.189510297	13141.42669	27.09098135	344604.655
2050	Male	1.179477979	15313.04532	1.549957706	19865.79942	1.189509431	13146.63568	27.06960957	344693.5108
2022	Female	0.581180652	6532.528017	0.711289296	8088.786553	0.542845453	6210.626342	13.18967149	150659.0731
2023	Female	0.577789833	6534.900312	0.69915733	8095.358612	0.541725034	6293.213038	13.05001829	151854.1168
2024	Female	0.574738095	6537.035378	0.68823856	8101.273465	0.540716657	6367.541064	12.92433041	152929.6561
2025	Female	0.571991532	6538.956938	0.678411668	8106.596832	0.539809118	6434.436288	12.81121132	153897.6415
2026	Female	0.569519625	6540.686341	0.669567464	8111.387863	0.538992333	6494.641989	12.70940414	154768.8284
2027	Female	0.567294908	6542.242804	0.661607681	8115.699791	0.538257226	6548.82712	12.61777767	155552.8966
2028	Female	0.565292663	6543.643621	0.654443877	8119.580526	0.53759563	6597.593738	12.53531385	156258.558
2029	Female	0.563490643	6544.904356	0.647996452	8123.073187	0.537000194	6641.483694	12.46109641	156893.6532
2030	Female	0.561868825	6546.039018	0.642193771	8126.216582	0.536464301	6680.984655	12.39430072	157465.2389
2031	Female	0.560409188	6547.060213	0.636971357	8129.045638	0.535981997	6716.535519	12.3341846	157979.666
2032	Female	0.559095516	6547.979289	0.632271185	8131.591788	0.535547924	6748.531298	12.28008009	158442.6505
2033	Female	0.55791321	6548.806458	0.62804103	8133.883323	0.535157258	6777.327498	12.23138602	158859.3364
2034	Female	0.556849135	6549.550909	0.62423389	8135.945705	0.534805659	6803.244078	12.18756137	159234.3538
2035	Female	0.555891468	6550.220915	0.620807465	8137.801848	0.53448922	6826.569	12.14811918	159571.8695
2036	Female	0.555029567	6550.823921	0.617723682	8139.472377	0.534204424	6847.56143	12.11262121	159875.6335
2037	Female	0.554253856	6551.366626	0.614948277	8140.975854	0.533948109	6866.454617	12.08067304	160149.0212
2038	Female	0.553555717	6551.855061	0.612450413	8142.328982	0.533717424	6883.458486	12.05191968	160395.0701
2039	Female	0.552927391	6552.294652	0.610202335	8143.546798	0.533509808	6898.761967	12.02604166	160616.5141
2040	Female	0.552361898	6552.690284	0.608179065	8144.642832	0.533322954	6912.5351	12.00275144	160815.8137
2041	Female	0.551852954	6553.046353	0.606358122	8145.629263	0.533154785	6924.93092	11.98179025	160995.1834
2042	Female	0.551394905	6553.366815	0.604719273	8146.517051	0.533003433	6936.087158	11.96292517	161156.6161
2043	Female	0.55098266	6553.655231	0.603244309	8147.316059	0.532867217	6946.127772	11.9459466	161301.9055
2044	Female	0.55061164	6553.914805	0.601916842	8148.035167	0.532744622	6955.164325	11.93066589	161432.666
2045	Female	0.550277722	6554.148422	0.600722121	8148.682365	0.532634286	6963.297223	11.91691325	161550.3504
2046	Female	0.549977196	6554.358677	0.599646872	8149.264842	0.532534984	6970.61683	11.90453587	161656.2664
2047	Female	0.549706723	6554.547906	0.598679148	8149.789072	0.532445612	6977.204477	11.89339623	161751.5908
2048	Female	0.549463296	6554.718213	0.597808197	8150.260879	0.532365178	6983.13336	11.88337056	161837.3827
2049	Female	0.549244213	6554.871489	0.597024341	8150.685505	0.532292786	6988.469354	11.87434745	161914.5955
2050	Female	0.549047038	6555.009437	0.59631887	8151.067668	0.532227634	6993.271748	11.86622665	161984.087
